# A Comprehensive Study of Cyber Attack Mitigation with the Exchange of Frequency Containment Reserves Control in a Multi-Infeed Direct Current Power System

**DOI:** 10.3390/s23041964

**Published:** 2023-02-09

**Authors:** Umar Fitra Ramadhan, Jaehyeong Lee, Minhan Yoon

**Affiliations:** Department of Electrical Engineering, Kwangwoon University, Seoul 01897, Republic of Korea

**Keywords:** cyber–physical, DoS attack, FDIA attack, HVDC reserve operation control, Korean electric power system, MIDC power systems, renewable energy, transient stability

## Abstract

By 2040, the Korean government aims for a penetration rate of 30–35% of the total power from renewable sources. Due to a lack of inertia, particularly in remote systems such as those on Jeju Island, these circumstances will reduce network stability. To maintain the diversity and unpredictability of RES penetration, HVDC systems with an exchange of frequency containment reserve control are utilized. An exchange of frequency containment reserves control (E-FCR) is one of the balancing arrangement concepts of HVDC systems. However, the development of E-FCR concepts is vulnerable to cyber attacks because this concept only considers one wide-area measurement for data exchange. This study established a simultaneous cyber attack operation, i.e., an attack was set at the same time as a contingency operation that affects the balancing arrangement between two regions. Multiple possibilities of cyber attack and mitigation operations were suggested according to their ability to access information in the MIDC system. Then, a cyber detection strategy was proposed through a normalized correlation concept to activate mitigation control that could enhance the frequency stability by adjusting the value of the ramp-rate deviation between two HVDC types. By simulating the Korean power system model that was implemented in PSS/E, along with a Python script, simulation results demonstrated that a cyber attack on missing data can cause severe low-frequency nadir responses, and the proposed methodology can practically detect and mitigate cyber attacks.

## 1. Introduction

A key characteristic of Korea’s energy industry is the predominance of fossil fuels in energy production [1]. Although the Republic of Korea amended its first Nationally Determined Contribution (NDC), which seeks to reduce global emissions, growth in the proportion of alternative energy sources to 20% in 2030 [2] as well as to 30–35% of the total in 2040 was reported [3]. A unique feature of RES penetration in Korea’s electricity grid is that it is irregularly dispersed and highly concentrated [4]. Transmission system operators (TSOs) may increase the flexibility in transfer capacity across asynchronous zones by using either AC or DC lines. The use of AC transmission lines has a flaw that makes the system more complicated and might negatively affect system dependability [5]. According to [6], a variety of parameters have been examined to distinguish AC and DC systems. These days, HVDC systems with reserve balancing strategies are employed to enhance the flexibility of renewable energy in each region and the flexibility of the transfer capacity for both regions. A study of the HVDC link’s performance with various balancing arrangement approaches in the MIDC system was suggested in our most recent work [7]. In addition, an enhanced effective short circuit ratio was introduced to help monitor the steady functioning of MIDC systems (ESCR) [8]. Three factors, i.e., the variable power system’s network size, a variation inside the HVDC line type, and the capacity of HVDC connections, make up the core constraint of the MIDC system. As a result, balancing arrangements may cause network constraints if the appropriate reserves are not performed well. 

In addition, according to [9], each HVDC type comprises three controller types, i.e., internal, external, and remote control. Moreover, the arrangement of balanced reserves with the MIDC system uses remote control. The aim of remote control in the MIDC system is to maintain the values of each HVDC set point, which are collected from real-time measurement data. Furthermore, the arrangement of reserve balancing through HVDC, especially the exchange of frequency containment reserve (E-FCR), has a high dependence on supervisory control and data acquisition (SCADA), as well as wide-area measurements, protection, and control (WAMPAC) platforms. As a result, the application of E-FCR control operations has led to increases in vulnerability to cyber attacks. The analysis of cyber threat modeling has been increasing in the deployment of smart grids, including HVDC technologies, especially after crucial incidents, e.g., the 2015 Ukraine blackouts that occurred after hackers used DoS and FDI attacks on the smart grid devices of the network system [10]. 

The fundamentals of power system cybersecurity have been widely analyzed [11]. The author of [12] proposed an impact analysis study of cyber attack operations on a new load–frequency control modeling scheme that consists of two transmission line types, i.e., AC and DC lines. Additionally, the security of the balancing arrangements in the MIDC system requires advanced analysis to develop multiple possible cyber-attack operations. This issue could be described through two main characteristics, i.e., the difference in HVDC interconnection capacity and HVDC line type. The work in [13] proposed an evaluation study of data injection attacks with several considerations, e.g., information access ability, blind information, and injection ability. Regarding these considerations, we could describe them as fewer requirement ability assumptions. Low-cost and almost blind data injection attacks could lead to significant large mismatches of power in both regions. From the perspective of Korean power system modeling, LFC with a special characteristic of the MIDC system is still needed to evaluate the effect of DoS and FDI attacks. If a mismatch of power occurs, the frequencies between the two regions will become a constraint. Therefore, a cyber attack might lead to severe consequences, such as the activation under frequency load shedding, i.e., the frequency nadir being lower than the actual value. 

Moreover, in this article, we analyzed the impact of different types of DoS and FDI attacks on the LFC system along with E-FCR balancing strategies. Furthermore, we proposed optimal strategies to determine mitigation control and to ascertain how realistic the cyber-attack is according to simultaneous operations, i.e., the design of the cyber attack was set similarly to the contingency operation. A recent study [13] only considered cyber-attack and defense strategies with a non-simultaneous operation, which cannot be applied in a real situation between two regions. Moreover, this study explained the novelty of normalized correlation concept detection and adaptive ramp-rate operation values as a mitigation control method. 

This article consists of several sections as follows. In Section 2, we describe frequency characteristics through the activation of E-FCR control along with a description of three stages of frequency feature responses, load–frequency characteristics, and the communication system in the MIDC system. Section 3 describes a mode of MIDC control that consists of two different main controls depending on the HVDC line type along with the WAMPAC application. Section 4 analyzes cyber–physical threat modeling that explains DoS and FDI attacks, detection methods, and mitigation control, considering the real application of the current measurement sensor. In Section 5, we introduce a simplified model of the Korean power system incorporating MIDC system characteristics, along with simulation results and a discussion of cyber attack detection and mitigation control through a proposed algorithm. The conclusion is described in Section 6. This paper concludes by analyzing the cybersecurity operation of a MIDC power system using PSS/E software and Python script. 

## 2. Frequency Characteristic by Exchange of Frequency Containment Reserves Control

We will present an overview of frequency quality goals in this section, along with the time and magnitude of frequency variation. To balance two asynchronous sectors in the Korean electricity system, we also offer the E-FCR model. Additionally, communication infrastructure and HVDC real-time data communication sensors are used to demonstrate the E-FCR concept’s operating principle.

### 2.1. Frequency Characteristic

**Figure 1:**
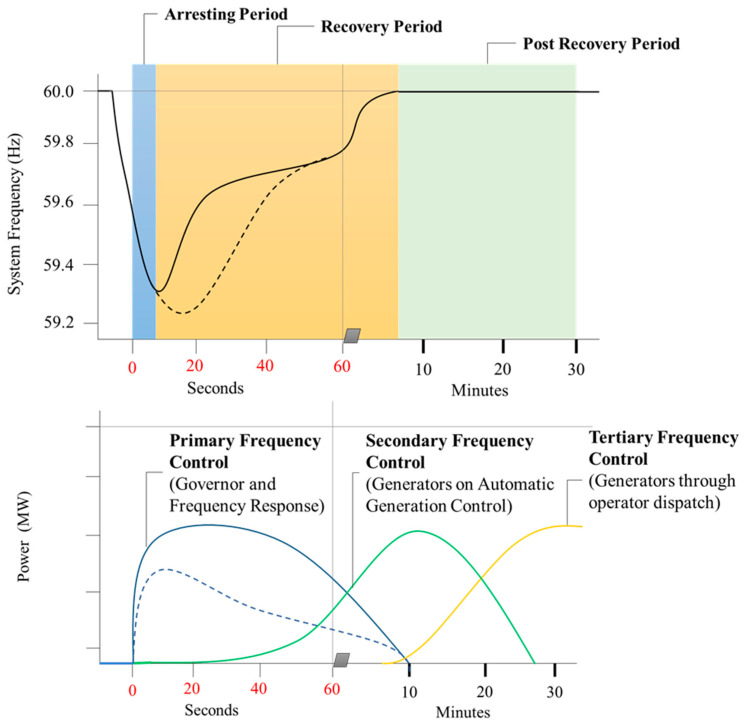
Feature of frequency response.

Because of uneven supply and demand, the disconnecting of multiple utilities and parts of the power system result in frequency deviation and fluctuation from the nominal value. Following a frequency deviation, the frequency responses are divided into three phases: the arresting phase, the recovery phase, and the post-recovery phase. The frequency response in the power system is depicted in Figure 1 [14]. The frequency shift in the power system is shown using the swing equation. The swing equation [15] is as follows:

(1) $$\frac{d}{\mathrm{dt}}f\;\times \;\frac{{2H}_{\mathrm{total}}}{f_{0}}=\frac{\Delta P}{S_{\mathrm{total}}}$$

where f is the system frequency, H_total_ is the system inertia constant, S_total_ is the amount of generator capacity, and f_0_ is the nominal frequency.

The inertia constant of the system is defined as follows:

(2) $$H_{\mathrm{total}=}\frac{\sum_{i=1}^{n}H_{i}{.S}_{i}}{S_{\mathrm{total}}}$$

where H_i_ is each generating type’s inertia constant, and S_i_ is the generator capacity. Addressing the stability of the power system, the frequency nadir and the Rate of Change of Frequency (RoCoF) value are the two most important considerations. The RoCoF and nadir value in the European power system are terminology used [16] to refer to the ENTSO-E requirements that are addressed during transient operations. According to [4], particular investigations are provided using the nadir frequency value for the greatest RES penetration in the Korean electrical system. Frequency stability is the retention of frequency values within standard limits. Discussions on the use of HVDC interconnectors for the control of the main frequency have been dominated by technical issues, such as setting the range of operation, power outages, and maximum discrete frequency variation of the HVDC connection. This study aimed to enhance the system’s frequency stability after a cyber attack occurs by promoting the main frequency response through employing several HVDC link cooperation techniques.

### 2.2. Load–Frequency Control

**Figure 2:**
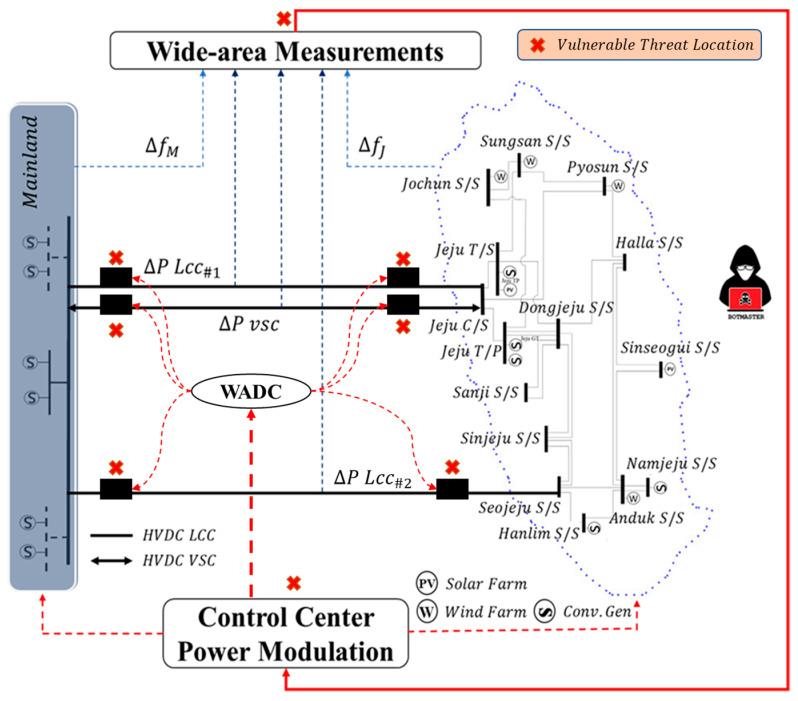
Several vulnerable point attack in MIDC system.

From the perspective of power system operation [16], The LFC is considered a closed-loop system that adjusts generator setpoints for active power output based on wide-area transmission data to maintain grid frequency and scheduled tie-line power between asynchronous zones. Using an HVDC connection, LFC’s primary goal is to ensure zero steady-state errors in a cross-border interconnected power system. The shifting from LFC blocks is necessary for HVDC operation, some of which increment or decrement power from the system. A speed droop depending on reserves of power and time iteration is enforced with primary control. Additionally, during the arresting and recovery phase, the main control will turn on the frequency containment reserve (FCR). Furthermore, the LFC model within MIDC systems often includes high-level control applications. As mentioned in Section 1, the reserve operation balancing arrangement that will be applied in this research is the E-FCR control. Moreover, the vulnerability of balancing configuration within two different size regions is explained in [17], which includes several potential cyber attack operations in the balancing arrangement system. In addition, Figure 2 depicts the specifics of the cyber attack that address the MIDC system’s several weak points particularly in red cross sign symbol.

### 2.3. Exchange of Frequency Containment Reserve (E-FCR)

**Figure 3:**
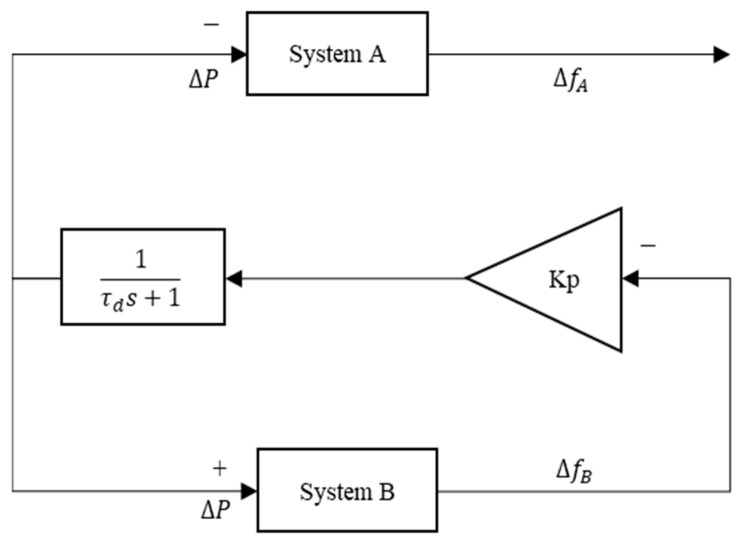
E-FCR control through HVDC operation.

The exchange of FCR control is another common way of managing HVDC to sustain a frequency comparable to the droop concept on HVDC. This study [18] presented the HVDC link between GB and CE utilizing multiple parameter gains values (Kp) as an exchange of the FCR control. The remarkable differences of exchange frequency containment reserves (FCRs), i.e., the input signal for E-FCR control only uses the frequency difference from one specific region [9,18]. As shown in Figure 3, a signal from System B, also known as Jeju Island, is used for the E-FCR control operation. Moreover, the frequency variation of Jeju Island (System B) is maintained at a value of 60 Hz during normal operation. However, the exchange of FCR control via HVDC is initiated when a contingency is applied. 

As shown in Figure 3, the topology control for the E-FCR control is uncommon because an LFC system network will only be able to adjust MIDC power based on the frequency deviation of one area. This characteristic makes the E-FCR method susceptible to frequency data sharing since it enforces just one broad area measurement, which is often left unsecured and makes the LFC system more open to cyber attacks. Additionally, the actual frequency droop response, i.e., 1%, of the Korean power system was discussed [19] to describe the various aspects of balancing arrangement schemes in the event of a cyberattack operation. On the other hand, in this study, the author approached similar droop values for each HVDC system. The main reason similar droop values are enforced is to increase awareness of power system operators in terms of cyber attack detection in the MIDC system. The gain value of Kp_(LCC/VSC)_ is applied based on the following equation:

(3) $${\mathrm{Kp}}_{\left(\mathrm{lcc}/\mathrm{vsc}\right)}=\frac{{\mathrm{Prating}}_{\left(\mathrm{lcc}/\mathrm{vsc}\right)}}{f_{0}\times {\;\mathrm{Droop}}_{\left(\mathrm{lcc}/\mathrm{vsc}\right)}\;\left(\%\right)}$$

where Prating_(lcc/vsc)_ is the maximum capacity of each HVDC line, i.e., 400 and 200 MW for LCC and VSC, and Droop_(lcc/vsc)_ is the real percentage value that is usually applied in the real HVDC frequency control in Korea’s power systems. A specific droop value for each HVDC system is enforced, e.g., 1% in LCC and VSC HVDC. The remarkable difference in Prating_(lcc/vsc)_ value will allow power system operators to adjust different gain value operations between two types of HVDC. In addition, through this situation, power system operators could maintain network security, especially when blind data and low-cost cyber attacks occur, i.e., attacks which only focus on one rectifier station.

### 2.4. Communication Interconnection System

**Figure 4:**
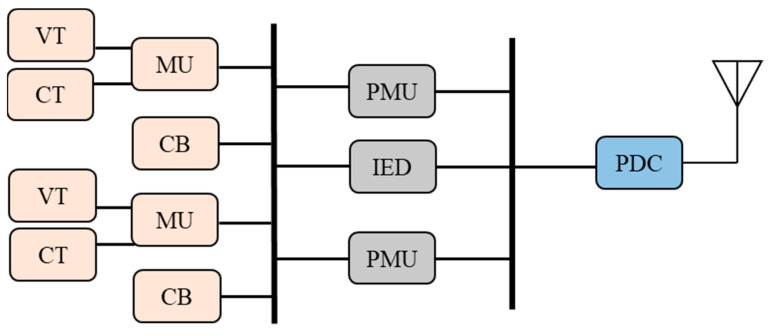
PDC and PMUs within a substation.

As depicted in Figure 4, Each converter station must establish a remote control interface for connection with the centralized frequency control optimization center, as described in [20]. The author of [16] suggested how to establish each HVDC converter station based on time synchronization (through GPS), which was mentioned in [21]. The main goal is to avoid problems with frequency stability and failure performance brought on by inaccurate local frequency signals. The WAMPAC platform’s essential building components are phasor measurement units (PMU) and phasor data concentrators (PDC) [22]. These components can measure voltage/current magnitude, phase angle, frequency, and other quantities at fast speeds and with great precision (30 to 120 time tagged samples per second), which are strategically located in substations. PDCs may connect with other PDCs within the several regions, as well as with nearby utilities, operation centers, and other PDCs to focus on synchro phasor data from various PMUs. In certain instances, mathematical techniques are used to interpolate the missing data. By using its processing power, PDC may carry out a wide range of other activities in addition to data concentration, including data archiving, access control, and data validation. Furthermore, there are three different color which shows the application of communication infrastructure level.

### 2.5. Communication Infrastructure System

**Figure 5:**
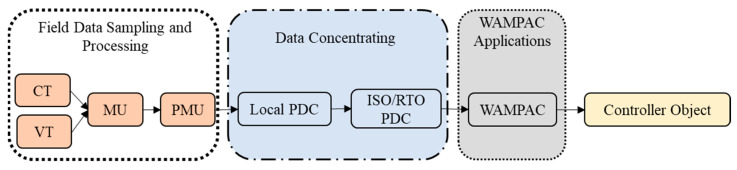
Phasor data processing stages in the WAMPAC Platform.

**Figure 6:**
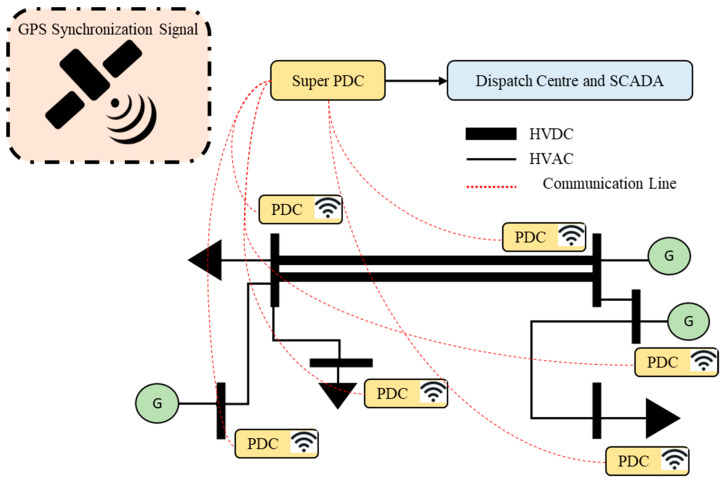
WAMPAC communication infrastructure.

As illustrated in Figure 5 and Figure 6, the communication infrastructure system of the WAMPAC platform is utilized [23]. The synchro phasor data are gathered via the networks and processed in phasor data concentrators (PDCs) to depict the electric power grid’s real-time operational characteristics. The communication networks integrate many PDCs that are geographically distributed at various substations using a variety of media, including metal wire, dedicated optical fiber, satellite, radio frequency, and microwave. Local PDCs may also interact with super PDCs at ISO/RTO to provide WAMPAC applications and situational awareness. The data may be enforced in wide area controls (WACs), software, and sophisticated data analysis tools to virtualize, evaluate, and operate each linked bulk power system as a whole [24]. Different WACs may be developed using the WAMPAC platform to provide higher performance that is not possible with conventional power systems. All PDCs should have reliable access to Coordinated Universal Time (UTC), which should be given via satellite and GPS time clocks, independent of the communication network. Regarding the difference color on communication infrastructure system is made according to hierarchy of communication infrastructure level.

## 3. Multi Infeed High Voltage Direct Current-Based WAMPAC Control

The WAMPAC platform through WADC is also used by another HVDC frequency reserve control application to enhance transfer rate and dynamic stability. Primary limitation oscillations on the tie lines connecting two quite remote region systems are the objective of WADC [25]. It is expected that the E-FCR concept through an HVDC system could effectively attenuate the oscillation between areas due to its exceptionally rapid power modulation capabilities via DC tie lines. This article introduced and discussed a typical HVDC-based WADC controller that is applied by FDI and DoS attacks.

### 3.1. MIDC Conrol Modeling

In this study, we proposed LCC and VSC models from PSS/E software for HVDC operation [26]. There are several LCC model types in PSS/E which are classified according to additional protection control. CDC4T has a standard operational characteristic that can be feasibly applied to the other models. Three distinct sorts of action controls in CDC4T modeling are described. The first action control is called normal regulation, which works by maintaining the rectifier and inverter’s set operating range value to a constant current or power. The second option is a momentary overriding of a dc converter, which maintains the performance as feedback control when AC system voltages are disrupted following malfunctions. The last action control involves modulating the DC power system response, which aims to eliminate the AC system’s rotor angle swings. Moreover, the correlation between frequency stability and the rotor angle was explored in [27].

On the other hand, VSC modeling is carried out using the VSCDYN control. The VSCDYN has three separate sorts of action via the controls, almost similar to LCC. The first action is reactive power control, also known as AC voltage control. The next is active power control, frequently referred to as DC voltage control, which is associated with two procedures, including as converter blocking and power ramping, if an unexpected event such as contingency takes place while the system is operating normally. The last action is a limitation of the current output, which seeks to maintain the current value while VSCDYN is applied.

### 3.2. MIDC Vulnerable Point According to WAMPAC Platform

**Figure 7:**
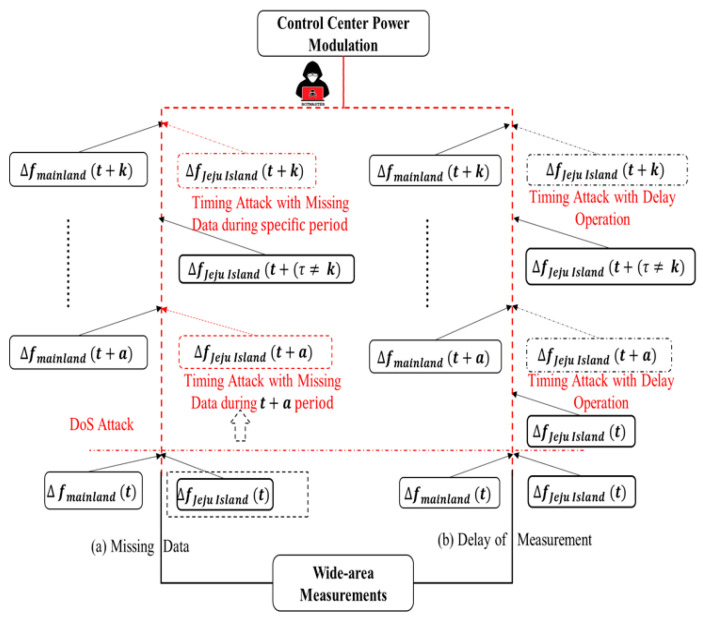
Illustration two types of DoS attack.

Technically, as seen in Figure 6, each level of data processing may be impacted by malicious and unintended cyber accidents. Figure 7 illustrates the idea that cyberattacks might undermine the WAMPAC platform [23]. The two most frequent threats are Denial-of-Service (DoS) and False Data Injection (FDI) attacks. So, at this point, the emphasis of this study is focusing modeling on those attacks.

DoS attacks could be enforced against the broad area platform that links each PDC which is located at substations and control centers. Even though DoS attacks cannot alter the measurement sensor interpretation included in PMU messages, this type of attack could result in significant time delays, packet losses, or even communication breakdowns by creating a large number of connection requests that overwhelm the target device’s processing power. Due to these circumstances, isolated PMUs depend on WAMPAC applications that affect HVDC controllers and cause them to operate less effectively or even negatively.

The data may be intercepted and altered by an FDI attack to accomplish the attacker’s goals. For instance, the measurements at the substation level collection may be immediately modified before being sent to PMU. Once delivered to PDC, the PMU phasor data may also be altered. Similar to this, control instructions created by WAMPAC applications and communicated across local and wide area networks may also be changed to directly impact the functioning of the control.

## 4. Cyber Physical System Threat Modeling

This section discusses threat modeling of a cyber-physical system in the MIDC system. As mentioned earlier, two attack types will be explained in this study e.g., denial of service and false data injection attacks.

### 4.1. Denial of Service Attack

Since denial-of-service attacks are amongst the biggest risks to collected data, they are primarily taken into consideration. Communication is often disrupted during a DoS attack, which could prevent measurement or modification information from accessing their appropriate locations. An attacker can use a variety of tactics to initiate DoS attacks. For instance, the attacker can saturate the network with traffic, target the routing protocols, infiltrate devices and stop them from providing data, and jam the communication channels [10].

As illustrated in Figure 7, denial-of-service attacks that utilize a fictitious time stamp include timing attacks to prevent communications among data both senders and recipients [28]. Inside the broad monitoring system for power grids, synchronized global time across PMUs apply a standard time reference from a satellite that contains time synchronization protocols, which might be hacked by either falsifying the time stamp or inserting a planned delay for malign objectives. Even if synchronization communications are encrypted and/or authenticated, the timing attack, which might just delay messages, may still endanger the controllers [29]. In this investigation, it was presupposed that attackers would purposefully lag behind the readings that the damping controller would receive. In particular, the attacker would alter the time stamp and induce a planned delay for the measurement of the HVDC transmission from the distant terminal. Furthermore, missing data attack operations are almost similar to false data injection attacks in terms of the malicious availability of data. However, the remarkable difference could be described through the timing attack operation itself. 

#### 4.1.1. Delay of Measurement Attack

**Figure 8:**
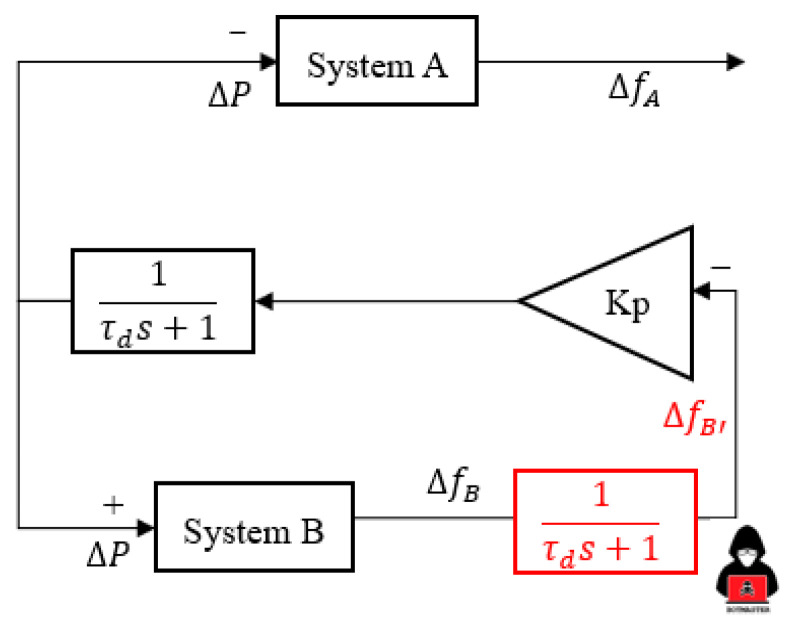
Denial of service attack in terms of E-FCR operation.

The communication system in HVDC through WAMPAC is important to maintain several integrations of the devices. As described in Section 3.2., there are many vulnerable points in WAMPAC platforms in which attackers can intercept the system. In this study, the author proposes one of the common denials of service in measurement devices. This attack could disturb the measurement system, which leads to misjudgment of the Exchange FCR concept and to inappropriate balancing arrangements between two asynchronous areas as well as coordination between two HVDC systems, e.g., VSC and LCC. As described in Figure 7, the delay of measurement attack is described through the characteristic of exchange FCR in Jeju Island. The red line shows a significant difference in the frequency response sequence, i.e., before and after an attack. In terms of delay of measurement, the frequency for both regions will not be similar, especially after Jeju Island is manipulated by the previous value of frequency measurement. This situation creates an unbalanced arrangement operation, e.g., the power modulation will not present actual frequency deviation and the ramp-rate of DC operation will decrease. According to this [30], the delay of measurement equation is expressed through Figure 8 especially in red color with the illustration of intruder, with the additional time delay operation before proceeding to E-FCR power modulation. 

#### 4.1.2. Missing Data Attack

**Figure 9:**
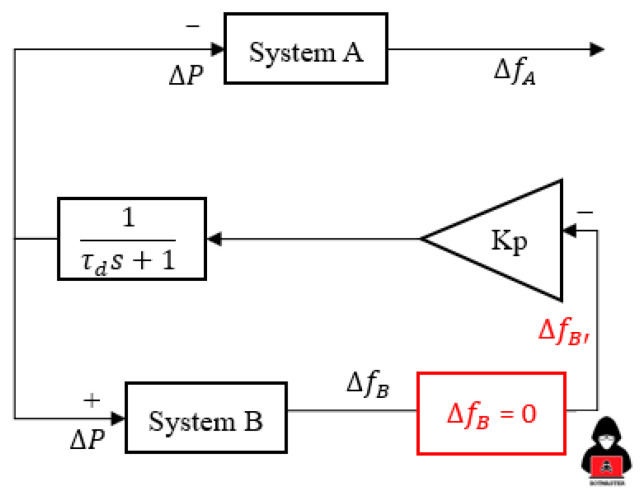
Missing data attack in terms of E-FCR operation.

This attack is categorized as denial of service because of its working principle similar to delay of measurement. The remarkable difference is expressed through the Jeju Island frequency system characteristic after an attack occurs. As illustrated in Figure 7, the false data of frequency are induced, i.e., with a similar frequency before an attack occurs. Furthermore, the detailed zero input of frequency signal is presented along with the block control of E-FCR operation in Figure 9 particularly in red color along with attacker illustration. This attack is significantly threatening due to the zero response of E-FCR operation if the contingency and attack appear at a similar time. Even though the data are restored, the coordination cannot be similar for the multi-infeed DC system as before due to the different values of active and reactive power judgment in another DC line. 

### 4.2. False Data Injection Attack

**Figure 10:**
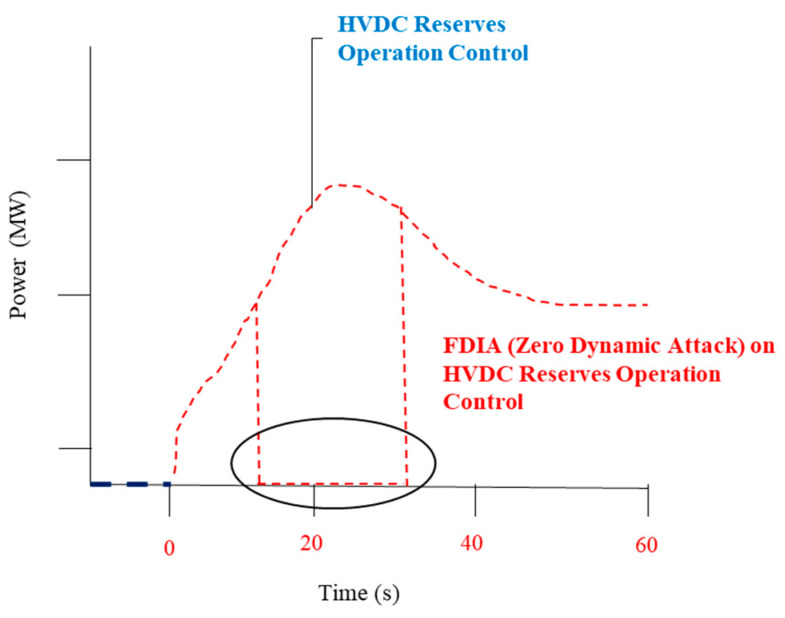
False data injection attack during E-FCR operation control.

As depicted in Figure 10, an FDI attack power signal reduces the power output until zero. This situation could be explained through FDI attacks that alter sensor network measurements and unintentionally inject erroneous data with faults to mislead operators or controllers. The exceptions are the timing attack and replay attack, which send a legitimate signal to the HVDC damping controller even while the timing is off, or if the signal is played again. An FDI attacks the measurement integrity system by substituting fraudulent measurement values while remaining undetected. Recent investigations have shown that if a certain number of sensor nodes are hacked, conventional authentication measures cannot stop an FDI attack. In this study [10], a mathematical equation was used to describe a characteristic of grid parameters during an FDI attack, especially at specific times.

### 4.3. Mitigation Control Method 

One of the many crucial factors affecting balancing arrangement control in terms of cyber security operation is the variation of HVDC interconnection capacity, as was stated in the introductory section. Additionally, we explored various HVDC interconnection types, which also constitute another crucial aspect of MIDC power system operation. Moreover, the various HVDC connectivity types could be used to perform cyber security detection of balancing cooperation concepts according to the droop value of E-FCR as an active power control management strategy. Nevertheless, a couple of references in the introductory section neglect to consider active power management regulation for cyber security detection and mitigation, notably in MIDC power systems.

**Figure 11:**
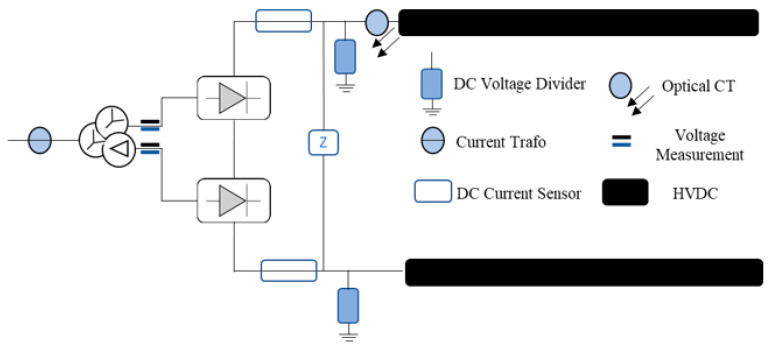
Measurement device placement in a HVDC converter station.

**Figure 12:**
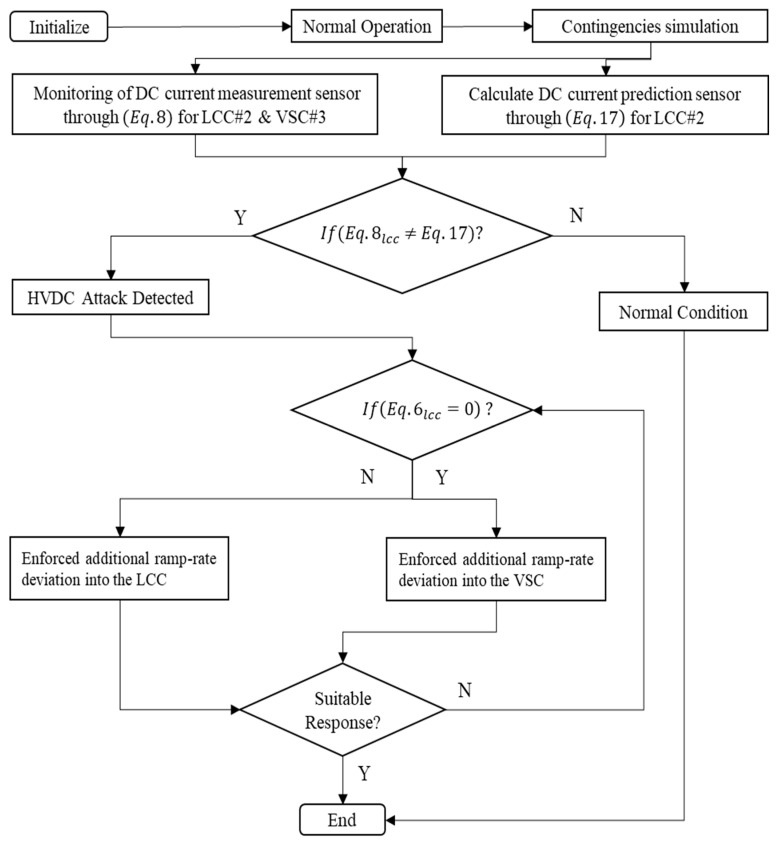
Algorithm of cyber attack mitigation control.

As illustrated in Figure 11 [9,31], the activation of active power control management through E-FCR control could be monitored through a DC sensor and due to the different specifications of voltage scheduling and rating capacity for each HVDC. The authors proposed a measurement based on per-unit conversion which could hopefully increase cyber attack detection in the MIDC system. Per unit, conversion could easily be achieved by using the calculation of Idc rate_(lcc/vsc)_ as described in Equation (4), as follows:

(4) $${\mathrm{Idc}\;\mathrm{rate}\;}_{\left(\mathrm{lcc}/\mathrm{vsc}\right)}\;(p.u.)=\frac{{\mathrm{Prating}}_{\left(\mathrm{lcc}/\mathrm{vsc}\right)}}{{\mathrm{Vsched}}_{\left(\mathrm{lcc}/\mathrm{vsc}\right)}\;}$$

where Vsched_(lcc/vsc)_ is the fixed voltage value of the HVDC system, i.e., 254 kV and 200 kV for the second LCC and VSC, respectively. The modulation power through E-FCR control could be calculated as follows:

(5) $$\Delta f_{b}=\;f-f_{0}$$

(6) $$\Delta P_{E-\mathrm{FCR}}{=\mathrm{Kp}}_{\left(\mathrm{lcc}/\mathrm{vsc}\right)}\times \Delta f_{b}$$

where Δf_b_ is the deviation between normal frequency along with the frequency when the contingency occurs. In addition, the author proposed a DC measurement that only reflected E-FCR activation, which could eliminate the initial power error calculation for each HVDC line. The calculation for DC current measurement based on per-unit conversion is described as follows:

(7) $${\mathrm{Idc}\;\mathrm{FCR}\;\mathrm{Measurement}}_{\left(\mathrm{lcc}/\mathrm{vsc}\right)}=\frac{\Delta P_{E-\mathrm{FCR}}\;}{{\mathrm{Vsched}}_{\left(\mathrm{lcc}/\mathrm{vsc}\right)}}\;$$

where ∆P_E-FCR_ is the addition of power modulation support through the multiplication gain value and frequency deviation.

(8) $${\mathrm{Idc}\;\mathrm{FCR}\;\mathrm{Measurement}}_{\left(\mathrm{lcc}/\mathrm{vsc}\right)}\;(p.u.)=\frac{{\mathrm{Idc}\;\mathrm{FCR}\;\mathrm{Measurement}}_{\left(\mathrm{lcc}/\mathrm{vsc}\right)}}{{\mathrm{Idc}\;\mathrm{rate}\;}_{\left(\mathrm{lcc}/\mathrm{vsc}\right)}\;(p.u.)}$$

where Idc FCR Measurement_(lcc/vsc)_ is the real current value of additional power modulation and Idc rate(_lcc/vsc)_ (p.u.) is the per-unit conversion value of the rating current. The ramp-rate deviation between LCC and VSC was used to create an adaptive droop control when mitigation operation was applied. The formulation is defined as follows:

(9) $$\Delta P_{\mathrm{rr}}=\left|{\mathrm{Idc}\;\mathrm{FCR}}_{\mathrm{lcc}}-{\mathrm{Idc}\;\mathrm{FCR}}_{\mathrm{vsc}}\right|\;(p.u.)$$

where Idc FCR_lcc_ (p.u.) is the per-unit conversion current value of additional power modulation through the LCC line and Idc FCR_vsc_ (p.u.) is the per-unit conversion current value of additional power modulation through the VSC line. As illustrated in Figure 12, the mitigation of low-cost and blind data injection attacks could be an approach using DC current prediction through a normalized correlation concept since E-FCR gain has different values between LCC and VSC types. The normalized correlation concept represents the comparable data between two actual DC current measurements, which has the goal of determining how similar the signals (DC currents) are. There are two functions that we want to compare, i.e., the actual DC current from two types, LCC and VSC, respectively.

(10) $$f/g\left(n\right)={\;\mathrm{Idc}\;\mathrm{FCR}\;\mathrm{Measurement}}_{\left(\mathrm{lcc}/\mathrm{vsc}\right)}\;(p.u.)$$

where Idc FCR Measurement_(lcc/vsc)_ (p.u.) is the per-unit conversion current value of additional power modulation through the LCC and VSC lines. In order to describe the detailed normalized correlation concept, we need to know the cross-correlation definition. Cross-correlation is the term that describes an inner product over two sensor data sequences [32].

(11) $$c\left(n\right)=f\left(n\right)\cdot g\left(n\right)=\overset{N-1}{\underset{i=0}{\sum }}f\left(n+1\right)g\left(i\right)$$

where f(n) is the function per-unit conversion current value of the LCC line and g(n) is the function per-unit conversion current value of the VSC line. Moreover, a normalized correlation concept is quite complex compared with cross-correlation due to a multiplication of inner sequences between two functions f(n) and g(n), which are defined as follows:

(12) $$p\left(n\right)=\;\frac{\sum_{i=0}^{N-1}\left[f\left(n+1\right)-\;\overset{-}{f}\left(n\right)\right]\left[g\left(i\right)-\;\overset{-}{g}\right]}{{\left\{\sum_{i=0}^{N-1}{\left[f\left(n+i\right)-\;\overset{-}{f}\left(n\right)\right]}^{2}\sum_{i=0}^{N-1}{\left[g\left(i\right)-\;\overset{-}{g}\right]}^{2}\right\}}^{1/2}}$$

where $\overset{-}{f}\left(n\right)=\frac{1}{N}\overset{N-1}{\underset{i=0}{\sum }}f\left(n+1\right)\mathrm{and}\;\overset{-}{g}=\frac{1}{N}\overset{N-1}{\underset{i=0}{\sum }}g\left(i\right)$. Moreover, this equation could be extended and defined as follows:

(13) $$p\left(n\right)=\frac{\sum_{i=0}^{N-1}\left[f\left(n+i\right)g\left(i\right)-\overset{-}{g}\right]\sum_{i=0}^{N-1}\left[f\left(n+i\right)-\overset{-}{f}\left(n\right)\right]\sum_{i=0}^{N-1}g\left(i\right)+N\left[\overset{-}{f}\left(n\right)\overset{-}{g}\right]}{{\left\{\sum_{i=0}^{N-1}f{\left(n+i\right)}^{2}-2f\left(n+i\right)\overset{-}{f}\left(n\right)\overset{-}{f}{\left(n\right)}^{2}\sum_{i=0}^{N-1}{\left|g\left(i\right)-\overset{-}{g}\right|}^{2}\right\}}^{1/2}}$$

(14) $$p\left(n\right)=\frac{\sum_{i=0}^{N-1}f\left(n+i\right)g\left(i\right)-\frac{1}{N}\sum_{i=0}^{N-1}f\left(n+i\right)\sum_{i=0}^{N-1}g\left(i\right)}{{\left\{\left\{\sum_{i=0}^{N-1}f{\left(n+i\right)}^{2}-\frac{1}{N}{\left[\sum_{t=0}^{N-1}f\left(n+i\right)\right]}^{2}\right\}\sum_{i=0}^{N-1}{\left|g\left(i\right)-\overset{-}{g}\right|}^{2}\right\}}^{1/2}}$$

In addition, the author proposed a cyber attack detection method through a DC prediction based on per-unit conversion, which can help power system operators acknowledge cyber-attack operations. Through Equations (9)–(14), we can find a DC prediction method according to the normalized correlation concept reflected by the inverse of gain value. Moreover, in this study, we focused on creating a prediction of LCC current, which is described as follows:

(15) $$r_{1}=\frac{1}{{\mathrm{Kp}}_{\mathrm{lcc}}}$$

(16) $$r_{2}=\frac{1}{{\mathrm{Kp}}_{\mathrm{vsc}}}$$

where Kp_lcc_ is the gain value of E-FCR control through the LCC line and Kp_vsc_ is the gain value of E-FCR control through the VSC line:

(17) $$f\left(\mathrm{nr}\right)=g\left(n\right)*\;\frac{{\;r}_{2}}{r_{1}}$$

where f(nr) is the prediction of the per-unit conversion current value through the normalized correlation concept in LCC line.

## 5. Case Study

This section further details the Jeju Island power system, which served as the subject of the simulation. Moreover, this section provides a detailed explanation of the cyber security detection and mitigation outcomes that cooperate with grid code requirements, such as frequency nadir. Additionally, comparison studies based on cyber attack modeling in the first HVDC interconnection are conducted. To determine power system stability standards after contingencies and the cyber attack period, this simulation time was set at 10 s. Additionally, two seconds after the simulation started, each contingency along with a low-cost and blind data injection attack study was implemented. As a result, the power system’s stability could be maintained by the HVDC link through a proposed methodology of mitigation, particularly when emergencies arose.

### 5.1. Jeju Island Power System

#### 5.1.1. Architecture and Description of the Electrical System

**Figure 13:**
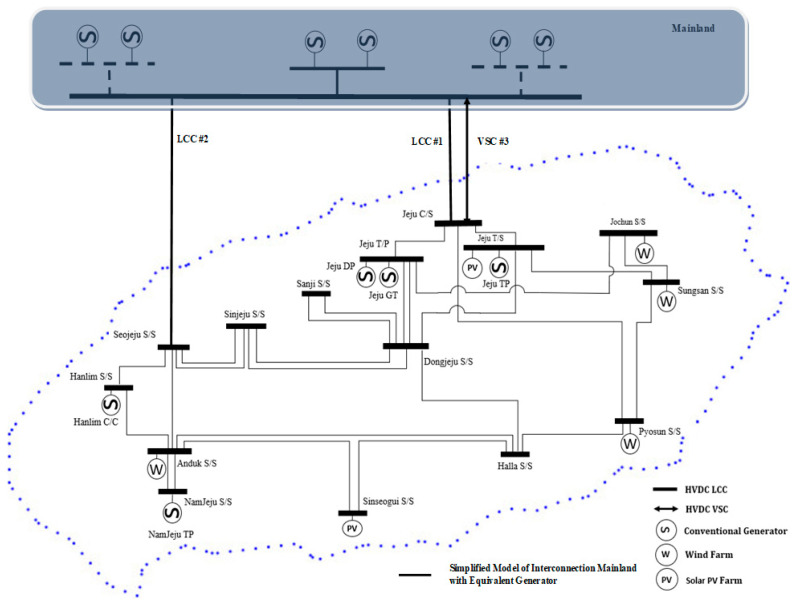
Jeju Island power system with HVDC configuration.

The architecture and description of the electrical system of Jeju Island are shown in Figure 13 [7]. Two criteria, including the generator type and the generator total capacity, are used to represent the mainland network system. For the generator model’s features, the largest percentage of generation in the Korean power system, i.e., coal-fired power generation, was used [1]. From the viewpoint of a power system researcher, since thermal power plants have a similar operating concept, thermal power plants might be represented as a GENROU, which is an abbreviation for the 2030 Eastern Interconnection Grid Report [33]. The capacity value of an equivalent generator on the mainland is shown in Appendix A, specifically in Table A1, in accordance with the general strategy of generating capabilities in the mainland power system. In addition, to ensure the correctness of the simulation findings, as mentioned in [33], the original capacities were replaced by an equivalent quantity that is 1.1 times the generator’s apparent output. The Jeju Island power system consists of four generators equally divided across 15 bus operating systems, and three HVDCs of two types, i.e., LCC and VSC. The first and second interconnections, which were constructed in 1998 and 2013, respectively, both employ the LCC type. Due to this, two LCC HVDCs may be installed at 180 kV/300 MW and 250 kV/400 MW, respectively. Furthermore, in this compressed model of the Korean energy system, the total demand on Jeju Island is 750 MW.

The capacity is 500 MW for typical HVDC operation and 250 MW for generation. According to several studies that were previously covered in the introduction, the VSC HVDC with a capacity of 200 kV/200 MW is a planned installation that can be addressed in the network system model of the Korean power system. Therefore, the first HVDC interconnection, established in 1998 with a capacity of 180 kV/300 MW, was utilized for contingency simulation studies. In consideration of [34], the highest limit of the HVDC operating reserve might be established using the rated power capacity. Additionally, the limit reserve operation of the second HVDC interconnection and VSC type was established to be completely accessible (250 MW to 400 MW, then 100 MW to 200 MW, respectively) in terms of frequency stability operations. 

#### 5.1.2. Considerations of MIDC Communications Systems for Cybersecurity 

The MIDC system consists of several HVDC lines with different rectifier stations. According to operational characteristics, this circumstance requires three types of control including internal, external, and remote control [9]. In the case of the Jeju Island network system, there will be unique characteristics since the rectifier station has a different switching mechanism control. Moreover, the most vulnerable transient stability operation could be seen through a mechanism of firing pulses with a thyristor in the LCC line. Additionally, the operation between the AC current of the converter and the voltage system is always lagging. Reactive power from the AC system should be enforced to remunerate lagging operations that could be monitored through a certain voltage level at each converter bus operation. On the other hand, VSC has more flexibility since reactive power capability could be enhanced by itself and does not require a certain value for switching the mechanism operation. As a result, internal control should be deployed to manage the basic switching mechanism for each HVDC type.

In terms of external control communication, there will be several hierarchical controls to establish appropriate voltage and power flow in the HVDC system. There are three main parameters that will be applied to this control, e.g., current, voltage, and the closed loop of power converter stations, which are usually managed through SCADA systems. As mentioned earlier in the previous section, three types of HVDC systems are introduced and which have different voltage and power rating values. Therefore, external communication could be used to maintain steady-state operation through the setting the desired value. 

Practically, the MIDC system consists of several one- or two-way communication technologies to adjust the balancing arrangement operation. For instance, the set point value of each HVDC line is controlled through remote control centers, while the measurement of real-time value is adjusted through SCADA to the control center. In addition, in this study, we approached the DC current measurement sensor as a monitoring parameter value. As mentioned earlier, most methods of reserve operation control of the balancing arrangement depend on PMUs through WAMPAC platforms. Due to the consideration of the possibility of multiple attacks at vulnerable points, cybersecurity attack modeling and mitigation control should be conducted, which could increase security and efficient power system operation. Moreover, the susceptibility of the WAMPAC platform to DoS and FDI attacks is depicted by the fact that many of the network system data are unauthenticated due to a large number of cryptographic authentications on devices.

### 5.2. Cyberattack Detection

As illustrated in Figure 12, an algorithm for cyber-attack detection through DC current measurement from E-FCR operation is proposed. Moreover, the normalized correlation concept for LCC#2 is also present to describe the prediction of DC current after the attack occurs on LCC#2. Furthermore, even though the attack only occurs on LCC#2, it will cause another effect, such as creating misjudgment active power from the VSC#3 line.

**Figure 14:**
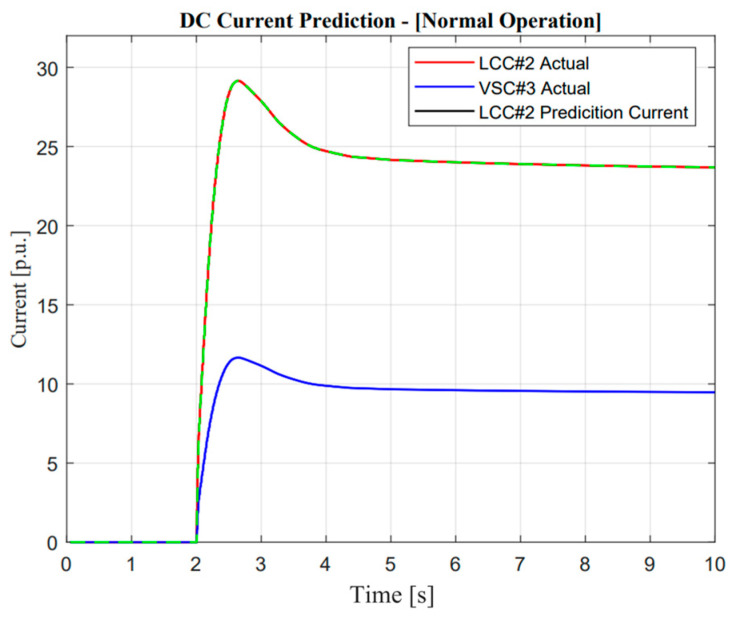
DC current prediction in normal operation.

**Figure 15:**
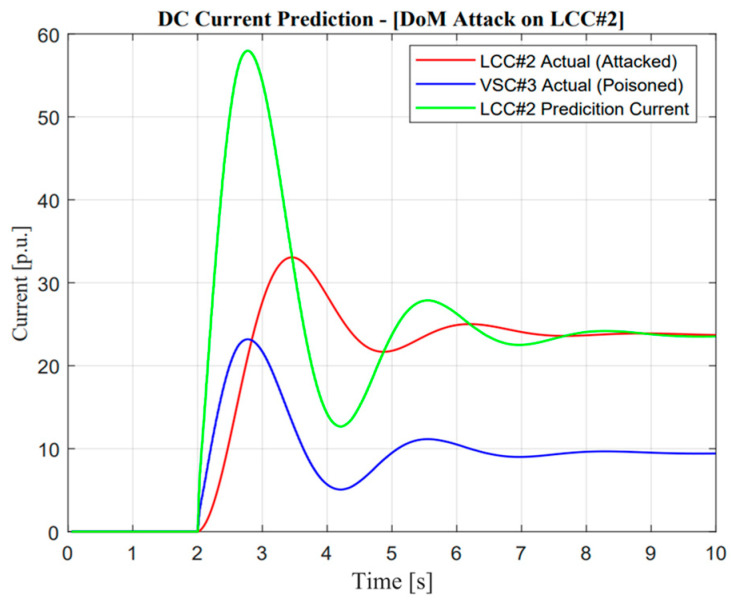
DC current prediction in delay of measurement attack operation.

**Figure 16:**
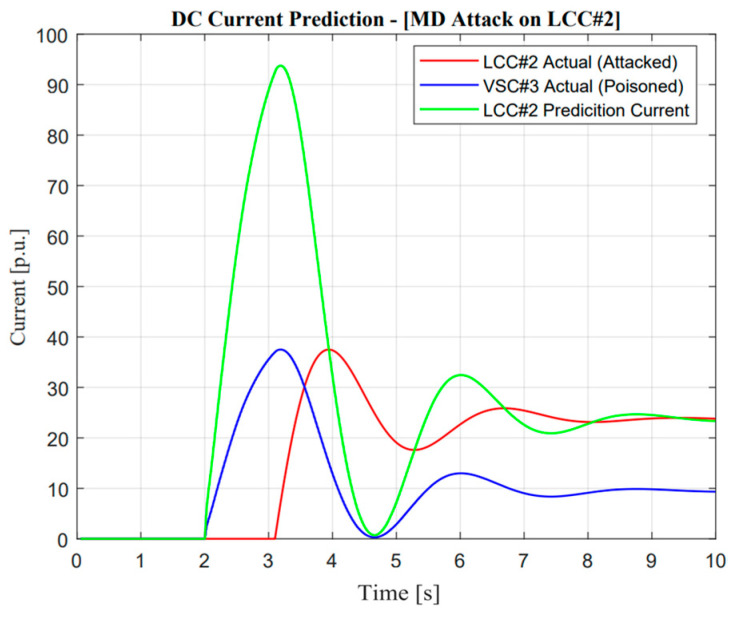
DC current prediction in missing data attack operation.

**Figure 17:**
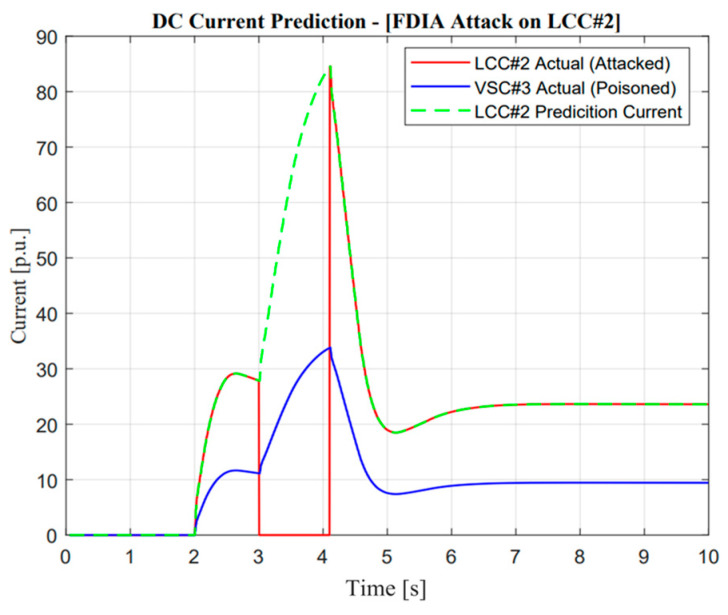
DC current prediction in false data injection attack operation.

As illustrated in Figure 14, DC current measurement detection from E-FCR operation was analyzed. In terms of normal operation, the prediction current showed an exactly similar response to the actual DC current from LCC#2. This shows that the proposed detection through normalized correlation concepts works well. The main reason why DC current prediction only focuses on LCC#2 is because when LCC#2 is attacked, it becomes a major threat for isolated power systems, especially for small-network-size balancing arrangements. In addition, it is also important to know the basic normal operational characteristics before we explore more cyber attack operations. As illustrated in Figure 15, Figure 16 and Figure 17, the response of DC current changes significantly after an attack occurs. 

As shown in Figure 15, when a delay of measurement attack occurs, the DC current from VSC#3 showed quite a significant difference. This situation was caused by an attack on LCC#2, in which the attackers change the input signal from the converter station reflected by the frequency measurement input signal in LCC#2 and create a delayed power transfer. 

As depicted in Figure 16, a missing data attack operation is more dangerous compared with a delay of measurement. This reason could be seen according to the zero response of the DC current, causing the attackers to change the input signal to zero several times. However, VSC#3 tried to support more power to maintain balancing arrangements similar to before the attack occurred. Due to this reason, we can observe a different ramp-rate value and maximum current compared with Figure 14 and Figure 15.

As described in Figure 17, DC current prediction works concerning the false data injection attack operation. Before the DC current was attacked, the actual and prediction currents showed a similar response. However, when intruders changed the signal to zero, the active current from VSC#3 suddenly changed the ramp rate in order to supply an imbalance after a zero dynamic attack occurred on LCC#2. This attack shows false data injection attacks are the major threatening operation in balancing arrangements. 

### 5.3. Denial of Service (DoS) Attack 

#### 5.3.1. Delay of Measurement (DoM) Attack

**Figure 18:**
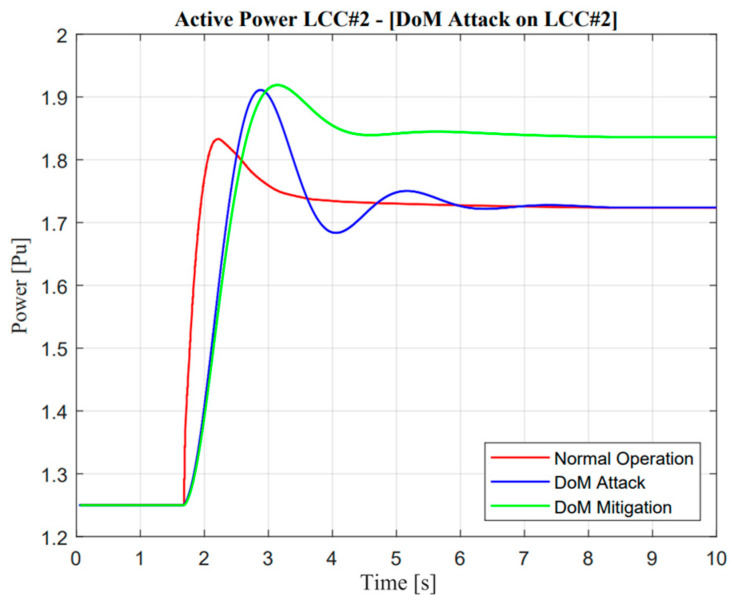
LCC active power response in the delay of measurement attack operation.

**Figure 19:**
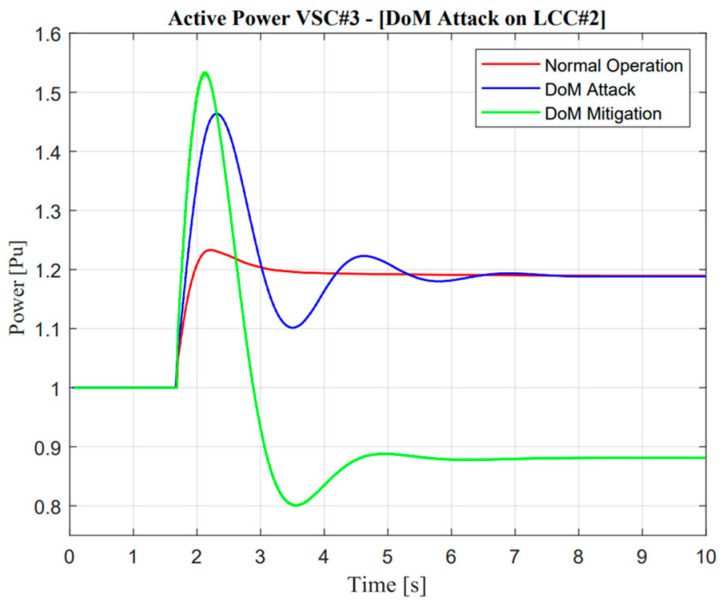
VSC active power response in the delay of measurement attack operation.

As illustrated in Figure 18 and Figure 19, the active power response from LCC#2 showed an exactly similar response to that reflected in the DC current operation. Furthermore, as mentioned in the previous section regarding cyber attack detection, DC current was used as the detection parameter. In a real application, the power system operator could easily monitor the DC current value through a sensor system. In terms of multi-infeed DC operations, the power system operator should monitor several lines, e.g., three lines in the Korean power system. Cyber attack detection should consider two-state operations, i.e., the current and prediction of the current value. In this study, DC current prediction was calculated using the normalized correlation concept. If there was a difference value between the current and prediction state, the attack operation was detected, and mitigation control should be approached with regard to the algorithm of cyber mitigation attack control. 

In the case of the DoM attack, as described in Figure 15, the actual current showed a different response from the prediction value. Naturally, power system operators should mitigate this problem. The proposed algorithm as described in Figure 12 could easily maintain the security of the balancing arrangement by adjusting the value of the ramp-rate operation. Moreover, a remarkable difference could be seen in mitigation control in the system. Mitigation control was activated in VSC#3 based on an adaptive droop application in a multi-infeed dc system. An adaptive droop calculation was calculated from the deviation of actual current according to Equation (9). Even though mitigation control was activated, there was complexity due to the characteristic of attack operation, especially at one rectifier station. Load–frequency control within the MIDC system majorly changed. This situation is exactly similar to those shown in Figure 18 and Figure 19; when the current through LCC was delayed, mitigation control automatically made an increment value of the current in the VSC line.

On the other hand, adjustment of additional power through VSC is also important, such as decrement, especially when additional power through the LCC line reaches a certain value and is consistent. In addition, the effect of the proposed methodology is described through the enhancement of frequency nadir value in the Jeju Island system. Moreover, the detailed description of the DoM attack and mitigation control is explained in the following section.

**Figure 20:**
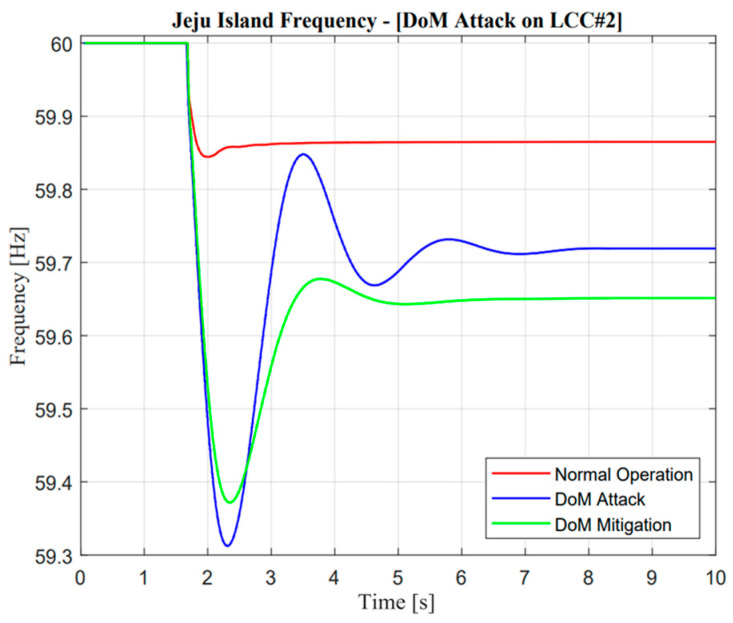
Jeju Island frequency in the delay of measurement attack operation.

**Figure 21:**
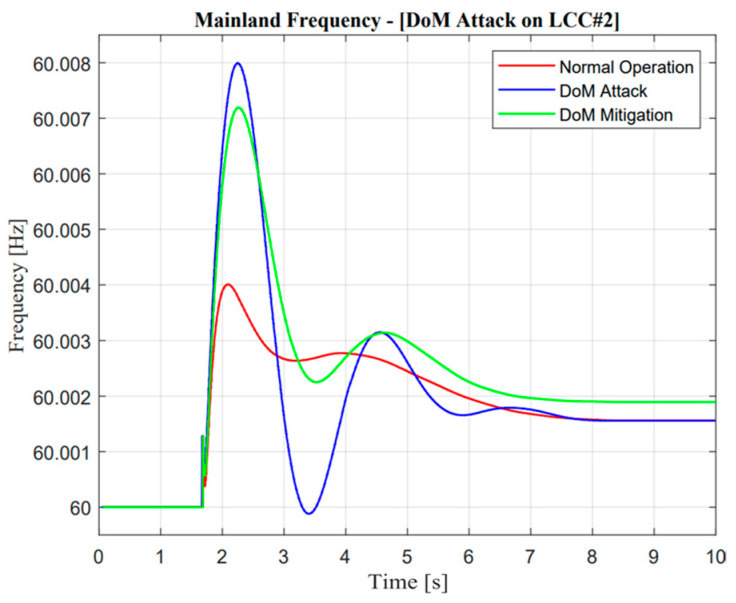
Mainland frequency in the delay of measurement attack operation.

As shown in Figure 20 and Figure 21, mitigation control is important when a delay of measurement attack has occurred. This situation could be determined through minimum frequency nadir, which must be applied in Jeju Island, i.e., 59.2 Hz. This frequency is important for standard evaluation because if the actual frequency value is lower than standard, load shedding must be active. However, a delay of measurement attack for 1.1 s could be tolerated due to the frequency nadir value remaining higher than 59.3 Hz. Moreover, the mitigation control showed exceptional improvement, which could improve the deviation of a frequency response by up to 0.82 Hz.

#### 5.3.2. Missing Data (MD) Attack

The real application of missing data attack and mitigation is completely similar to DoM attacks, which use detection from DC current sensors as a measurement. In the event of a missing data attack, as shown in Figure 16, the actual current showed a significantly different response from the prediction value, i.e., there will be no current output in actual measurement, especially when the contingency occurs. Instinctively, the power system operator should mitigate this problem to avoid large mismatches of balancing arrangement control. A similar explanation of the proposed algorithm in the previous section would increase balancing arrangement stability. 

Furthermore, a noticeable difference could be seen in VSC current response. As mentioned in the previous study, mitigation control was enhanced through the VSC line reflected in the ramp-rate deviation value, which is calculated from the actual current between two lines as described in Equation (9). Moreover, when missing data attack mitigation control is activated, there will be complexity due to the characteristic of different maximum power rating values for each line. Adjustment of additional power through VSC is also important, such as increment, especially when additional power through the LCC line is limited at a certain value, i.e., zero power response. The effect of the proposed methodology is shown through frequency nadir enhancement value in the Jeju Island system. 

**Figure 22:**
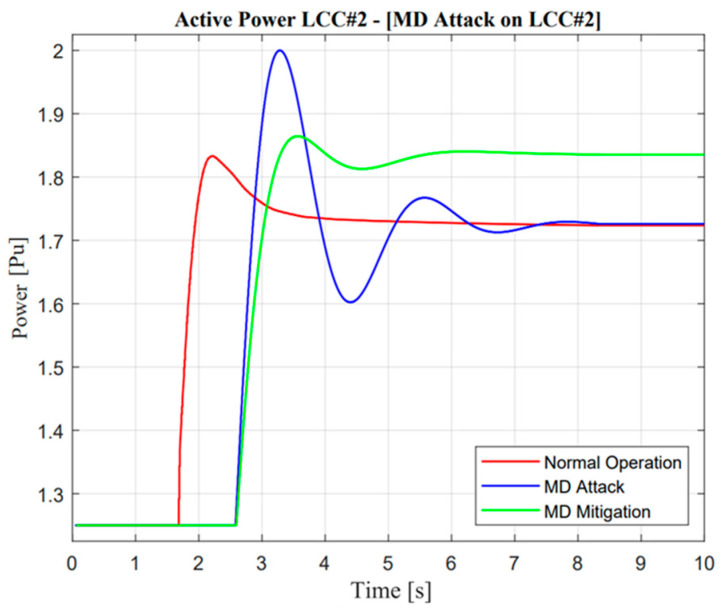
LCC active power response in the missing data attack operation.

**Figure 23:**
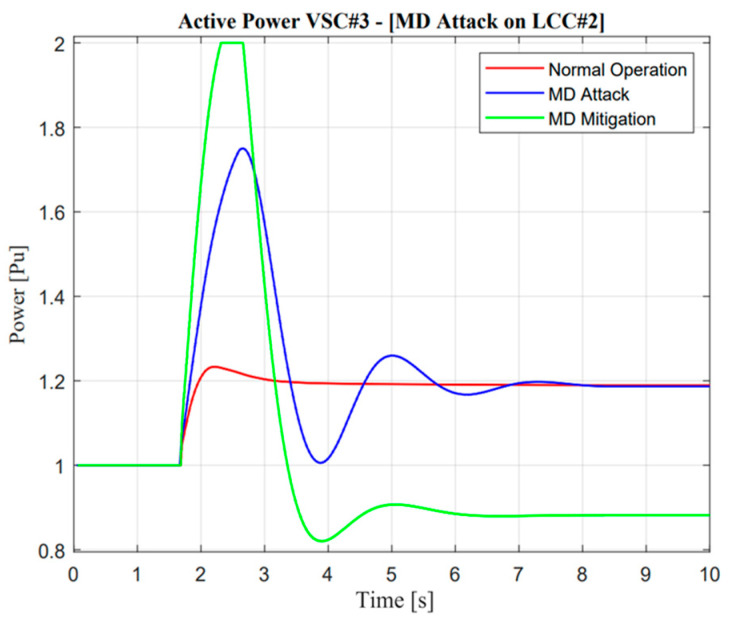
VSC active power response in the missing data attack operation.

As illustrated in Figure 22 and Figure 23, the active power from LCC and VSC showed remarkable differences in terms of balancing arrangement operations. When missing data occur and create zero response after a contingency operation, this situation showed the worst condition because there were huge differences in the power balancing of the HVDC line after the first HVDC interconnection trip. However, balancing arrangement is not always the mismatches of the amount of active power. The fast response is also important to maintain the frequency of nadir operation. This is clearly explained through active power description; when the active power response is attacked, the frequency nadir will lower than 59 Hz and the load shedding must be activated. However, when mitigation operation is activated, the adaptive response from VSC#3 control cannot help much to avoid load shedding. This situation could describe the maximum power rating in the VSC#3 line.

**Figure 24:**
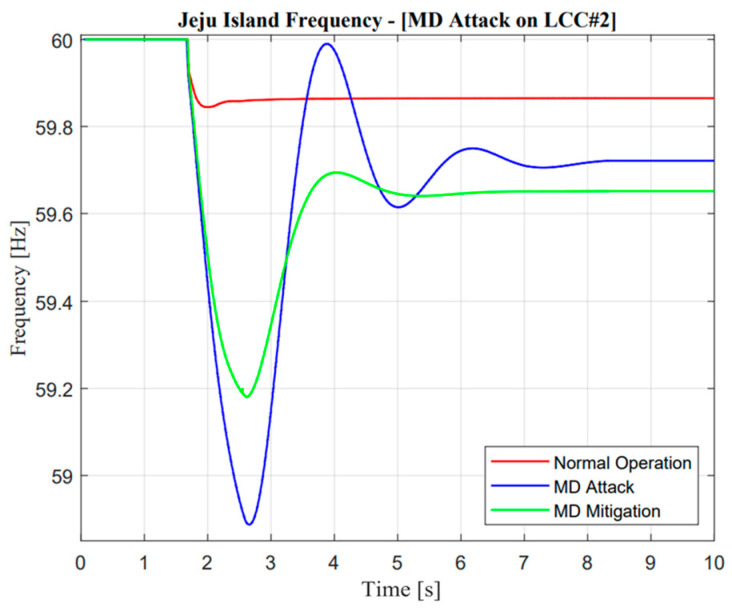
Jeju Island frequency in the missing data attack operation.

**Figure 25:**
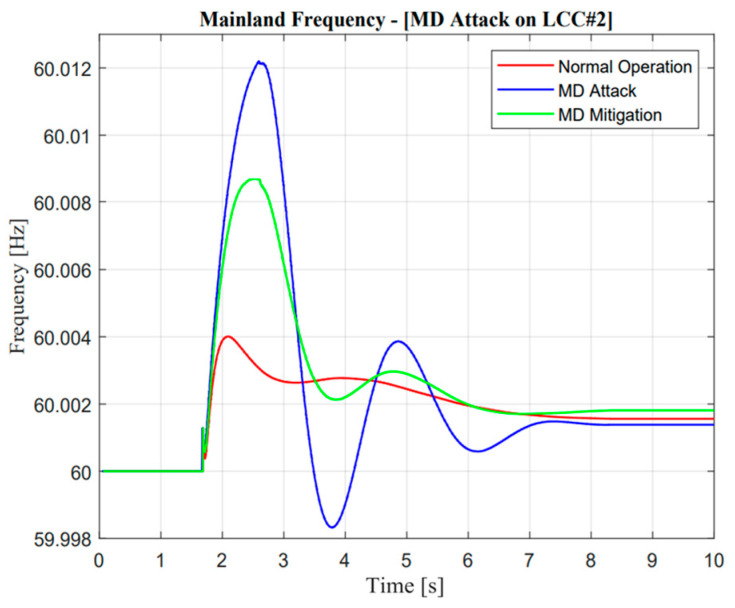
Mainland frequency in the missing data attack operation.

As illustrated in Figure 24 and Figure 25, it is important to explore mitigation control more advanced with other ancillary services when a missing data attack has occurred. As mentioned earlier, even though the mitigation operation is activated, the frequency is still lower than the standard value, i.e., 59.185 Hz due to the limitation of the maximum power rating in the VSC#3 line.

### 5.4. False Data Injection (FDI) Attack

Concerning the real application of false data injection attacks and mitigation operations completely similar to DoM, missing data attack DC current sensors as a measurement method are used for cyber attack detection. Concerning missing data attacks, as shown in Figure 17, the actual measurement current shows several significantly different responses from the prediction value. As mentioned in section two, there are several stages of frequency response features such as arresting, recovery, and post-recovery period. Moreover, a false data injection attack occurred during the recovery period, signifying the constant zero additional current value through the LCC line. On the other hand, the current measurement and prediction showed an identical response at the arresting period. This circumstance verifies that the detection of normalized cooperation concepts was performed well. As a result, the power system operator should alleviate this problem to avoid large mismatches of balancing arrangement through the proposed algorithm. 

Nevertheless, the VSC current response showed a noticeable difference. As was noted in the previous analysis, the VSC line improved mitigation control as indicated by the ramp-rate deviation value, which was obtained using the actual current flowing between two lines as described in Equation (9). Furthermore, because each line has a varied maximum power rating, there will be complications when the missing data attack mitigation mechanism is activated. When additional power through the LCC line is restricted to a specific amount, such as zero power response, adjustment of additional power through the VSC is equally crucial, as it is also incremented. The frequency nadir improvement value in the system around Jeju Island illustrates the impact of the proposed methodology.

**Figure 26:**
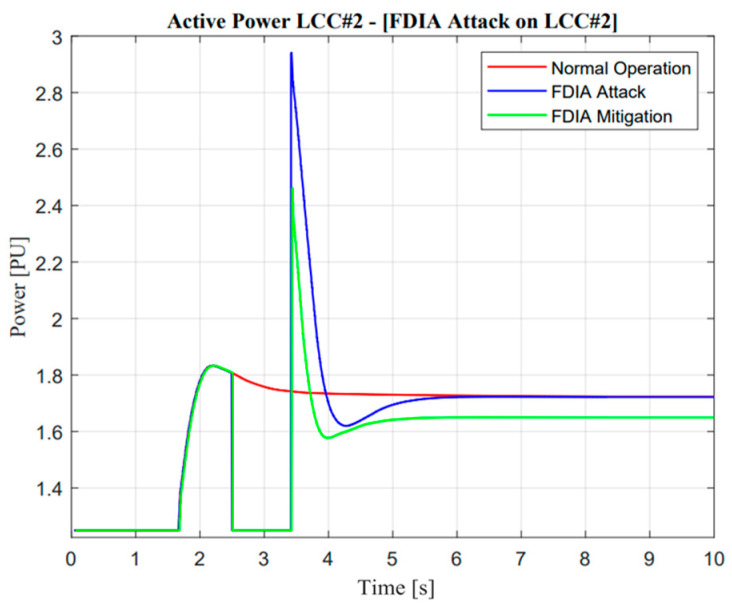
LCC active power response in the false data injection attack operation.

**Figure 27:**
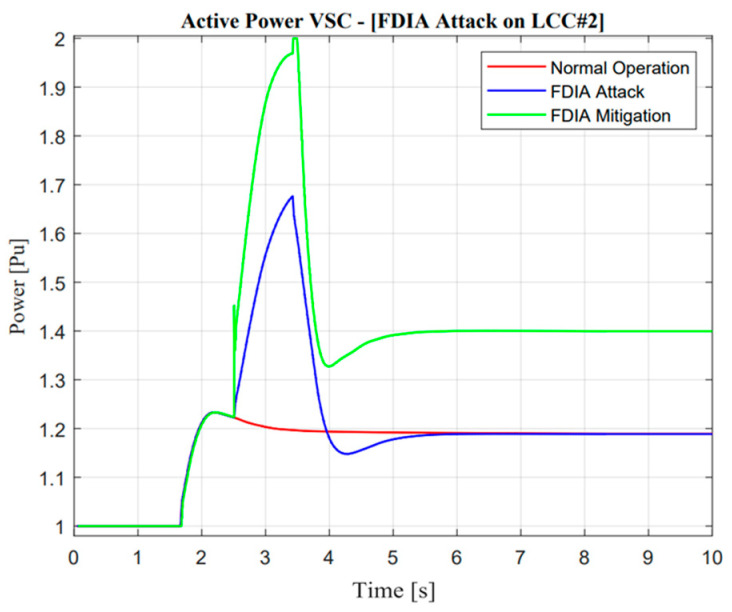
VSC active power response in the false data injection attack operation.

As illustrated in Figure 26 and Figure 27, the active power response will reduce to zero when false data injection is applied at the specified time. This attack operation shows a similarity with a missing data attack. However, this attack showed slight differences because the E-FCR operation was already active at the temporary time. On the other hand, there was a significant negative effect of this attack; this situation could be explained through mitigation operation response. When adaptive control was activated, it was useless due to the limitation of active rating power capacity in the VSC#3 line. These remarkable differences could be explained only by different ramp-rate values when attack and adaptive control through VSC#3 line are applied. In addition, a poisoned signal from the VSC#3 line showed good performance due to zero response when an FDI attack suddenly occurred.

**Figure 28:**
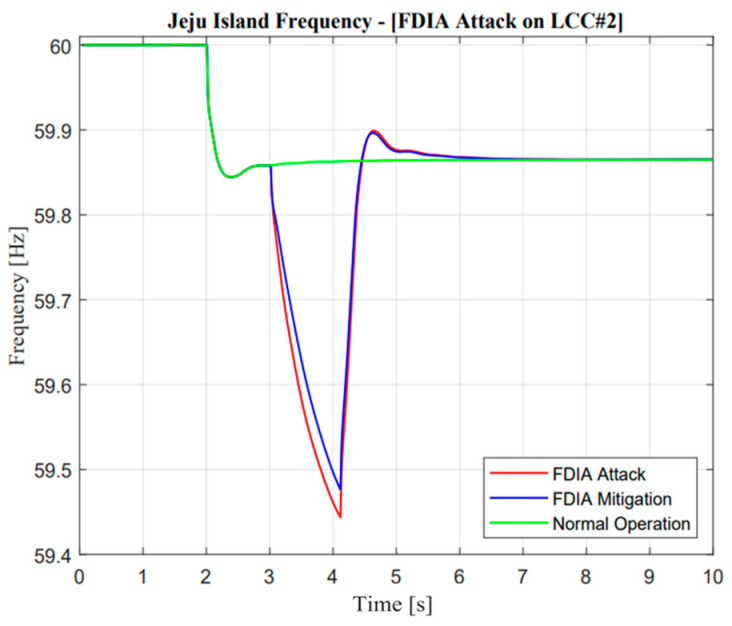
Jeju Island frequency in the false data injection attack operation.

**Figure 29:**
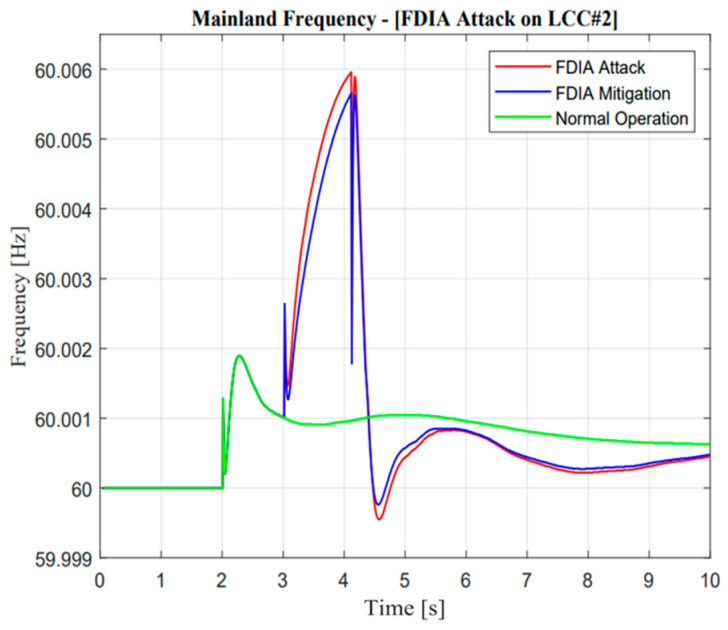
Mainland frequency in the false data injection attack operation.

As illustrated in Figure 28 and Figure 29, the response of frequency nadir in Jeju Island showed a better response compared with missing data attacks. This situation could be reflected in temporary power transfer from E-FCR. Even though power transfer was suddenly lost between two asynchronous areas, the frequency maintained a higher than minimum frequency nadir value, i.e., 59.4 Hz. However, when the attacked time was cleared, the steady-state value of power and frequency response was similar to normal operation. On the other hand, this situation cannot be described as a missing data attack due to large mismatches in terms of balancing arrangement operations.

## 6. Conclusions

The purpose of this article was to examine the security and capacity enhancement of E-FCR control from cyberattacks, particularly DoS and FDI attacks. As a point of vulnerability for the low-cost and blind data injection attack, we modeled the simplified MIDC system under DoS and FDI attacks. Two different types of DoS attacks were specifically suggested to assess their impact on the three stages of feature frequency responses. The emphasis was on improving the steady-state and frequency nadir contributions in terms of the balancing arrangement along with frequency stability control. Additionally, different power system sizes, such as those on Jeju Island and the mainland, were examined during contingency and cyber attack operations.

The first contribution involves creating a normalized correlation concept as a method of cyber security detection based on the real DC current measurement in the MIDC system. Regarding the theoretical normalized correlation concept, simulations on three different types of attack were used to conduct a comparative study of three states of cyber attack operations, such as a normal, attack, and mitigation operation. The second contribution is described as an improvement to the mitigation control using the adaptive ramp-rate value. Without mitigation measures, network security may be compromised, for example, if the frequency nadir drops below a predetermined value and unintentional load shedding occurs. According to the study, the MIDC power system’s response to a cyber attack on missing data was at its worst because there was no power available when the contingency occurred. The energy storage system (ESS) that is available in the Jeju Island power system should be taken into account along with other ancillary services when exploring development research. The specified mitigation strategy for a number of ancillary services may also be improved by the authors, as well as the system’s stability and dependability.

## Data Availability

Not applicable.

## Abbreviations

The following abbreviations are used in the manuscript.

CB	Circuit breaker
CT	Current transformer
DC	Direct current
DoS	Denial of service
DoM	Denial of measurement
E-FCR	Exchange of frequency containment reserve
FDIA	False data injection attack
GPS	Global positioning system
HVAC	High-voltage alternating current
HVDC	High-voltage direct current
IED	Intelligent electronic devices
ISO	Independent system operator
LCC	Line commutated converter
LFC	Load–frequency control
MD	Missing data
MIDC	Multi-infeed direct current
MU	Measurement unit
PDC	Phasor data concentrators
PMU	Phasor measurement unit
RTO	Regional transmission organization
SCADA	Supervisory control and data acquisition
VSC	Voltage sourced converter
VT	Voltage transformer
WAMPAC	Wide-area measurements, protection, and control

## Appendix A

Using a modeling approach of the Korean power system, the author examines the generator’s parameter information in this section. The sizeable generator capacity on the mainland and Jeju Island are shown, as mentioned in Section 4. The size of the network system is one of the difficult parameters to balance in the system architecture, as shown in the table. Furthermore, because it is the best model for thermal power plant operation, the electricity system on Jeju Island uses a generator based on the GENROU type. The Jeju Island power system operates each generator in two-unit mode, as shown in Figure 8. Additionally, Jeju Island’s generator will automatically adjust its operation to meet peak load requirements for the island’s power grid.

**Table A1:** The simplified model’s generator model capabilities.

Location	Model	Pgen (MW)	Pmax (MW)	Pmin (MW)	Inertia Value (H)	Capacity (MVA)
Mainland G-1	GENROU	250	36,500	180	3.027	36,500
Mainland G-2	GENROU	250	36,500	180	3.027	36,500
Jeju TP#2	GENROU	60	75	42	5.4	97.06
Jeju TP#3	GENROU	60	75	42	5.4	97.06
S-Jeju TP#3	GENROU	65	100	50	5.93	130
S-Jeju TP#4	GENROU	65	100	50	5.93	130
